# *In Silico* Methods Applied in Drug and Pesticide Discovery

**DOI:** 10.3390/molecules31162899

**Published:** 2026-08-20

**Authors:** Ana Borota, Diana-Ionela Popescu Stegarus, Gildardo Rivera Sanchez

**Affiliations:** 1 Coriolan Dragulescu Institute of Chemistry of the Romanian Academy, 24 M. Viteazu Avenue, 300223 Timisoara, Romania; 2 National Research and Development Institute for Cryogenic and Isotopic Technologies—ICSI Ramnicu Valcea, 240050 Ramnicu Valcea, Romania; diana.stegarus@icsi.ro; 3 Laboratorio de Biotecnología Farmacéutica, Centro de Biotecnología Genómica, Instituto Politécnico Nacional, Boulevard del Maestro s/n, Col. Narciso Mendoza, Reynosa 88710, Tamaulipas, Mexico; giriveras@ipn.mx

## 1. Introduction

In recent decades, the integration of computational approaches into drug discovery has transformed the identification and optimization of biologically active molecules. Molecular modelling, virtual screening, quantitative structure–activity relationship (QSAR) analysis, molecular dynamics, machine learning, cheminformatics, and systems biology now support multiple stages of the discovery process. These techniques enable the rapid analysis of extensive chemical and biological datasets, facilitate the exploration of ligand–target interactions, and help researchers prioritize compounds for synthesis and experimental evaluation [1,2].

Although computational methods are firmly established in pharmaceutical research, their application to pesticide discovery remains comparatively less developed [3]. Nevertheless, *in silico* approaches offer considerable opportunities for agrochemical innovation by supporting target identification, activity prediction, selectivity assessment, lead optimization, and the design of compounds with improved environmental and toxicological profiles [1]. These capabilities are particularly relevant in the context of food security, environmental sustainability, and the management of resistance to existing pesticides [4,5].

*In silico* methods are especially valuable during the early stages of discovery. Virtual screening can be used to evaluate large compound libraries [6,7], while docking and structure-based design can provide hypotheses concerning ligand orientation and molecular recognition [8]. QSAR and machine learning models can relate chemical structure to biological activity, whereas molecular simulations may offer insight into conformational flexibility, binding stability, and the determinants of selectivity [9]. By reducing the number of compounds requiring synthesis and testing, these approaches can contribute to more efficient and focused discovery workflows [10,11].

However, computational predictions cannot replace experimental science. Their reliability is influenced by the quality of the molecular and biological data, receptor preparation, ligand protonation and tautomeric states, the treatment of molecular flexibility, the scoring function, and the applicability domain of the model. In vitro, biochemical, analytical, and, where appropriate, in vivo investigations remain essential for confirming predicted activity, identifying false positives, assessing selectivity, and refining computational protocols. The integration of computational and experimental methodologies therefore provides not only greater reliability but also a deeper understanding of the chemical and biological mechanisms underlying activity [12].

Recent advances in artificial intelligence, big-data analytics, and high-performance computing continue to expand the capabilities of *in silico* methodologies. These technologies facilitate analyses of complex relationships between molecular structure, biological activity, pharmacological response, and environmental behaviour. At the same time, they increase the need for transparent reporting, reproducible workflows, independent benchmarking, and careful assessment of model uncertainty [13].

## 2. Overview of the Special Issue

**Figure 1:**
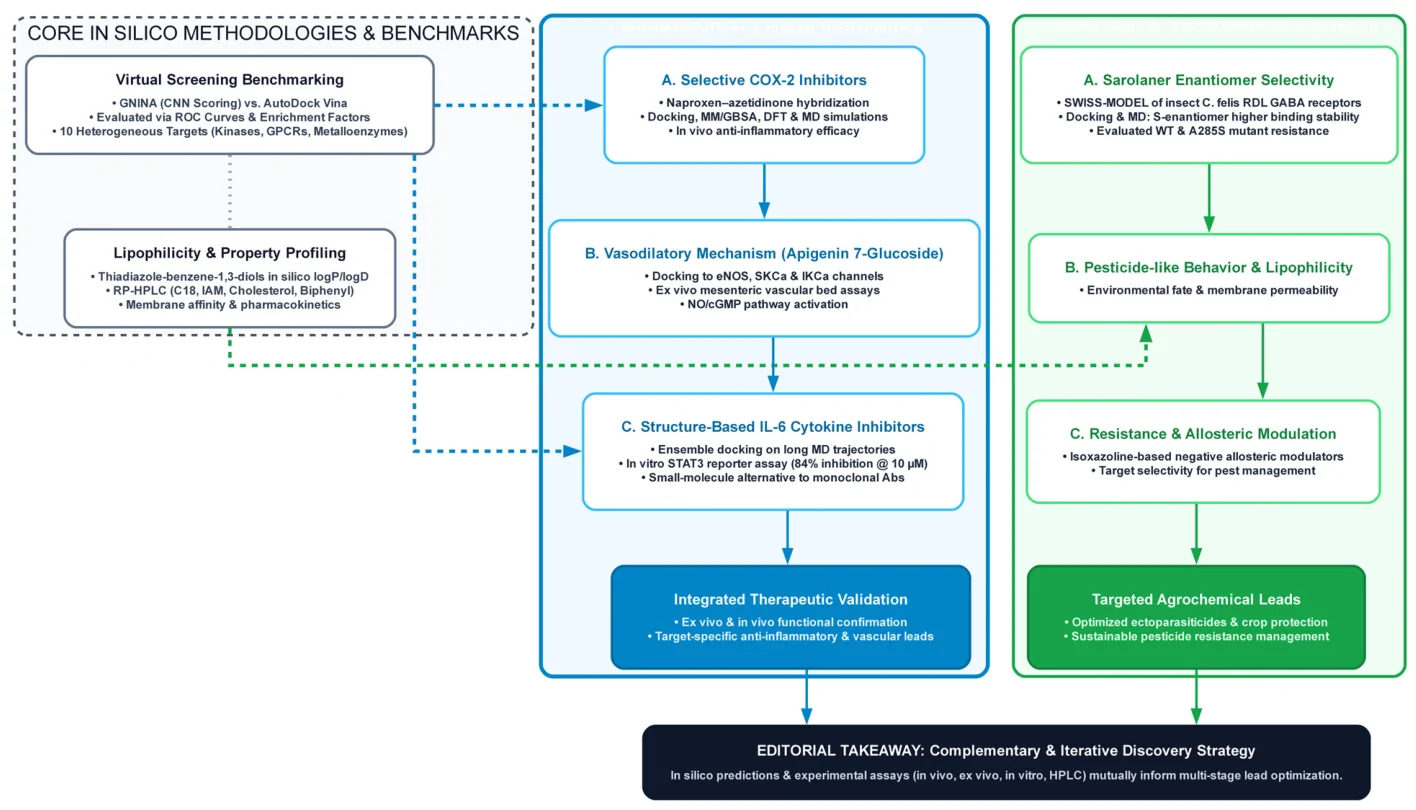
Complementary *in silico*–experimental strategy for drug and agrochemical lead optimization (*In Silico* Methods Applied in Drug and Pesticide Discovery—3rd Edition). Graphical abstract of the Special Issue. Gray boxes represent core *in silico* methodologies and benchmarks; blue boxes represent pharmaceutical and human therapeutic applications; green boxes represent agrochemical and ectoparasitic innovation. Solid arrows indicate the workflow progression within each research domain, while dashed arrows indicate cross-domain connections and feedback loops between core methodologies and specific applications. Dark-filled boxes indicate integrated outcomes and final leads.

This Special Issue, entitled “*In Silico* Methods Applied in Drug and Pesticide Discovery—3rd Edition”; https://www.mdpi.com/journal/molecules/special_issues/709L062VN1 (accessed on 11 August 2026), brings together six contributions that illustrate the diversity of current computational strategies and their integration with experimental research. The articles address methodological benchmarking, structure-based inhibitor design, anti-inflammatory chemistry, vascular pharmacology, heterocyclic compounds, and selective antiparasitic agents. Together, they demonstrate that computational methods can be used not only to rank compounds but also to generate mechanistic hypotheses concerning activity, selectivity, stereochemical recognition, and binding stability (Figure 1).

### 2.1. Selective COX-2 Inhibition with Naproxen–Azetidinone Hybrids

The article “Design, *In Silico*, and Experimental Evaluation of Novel Naproxen–Azetidinone Hybrids as Selective COX-2 Inhibitors” (Contribution 1) presents a comprehensive medicinal chemistry study in which naproxen is hybridized with two azetidinone scaffolds to improve cyclooxygenase-2 selectivity and mitigate adverse effects associated with non-selective COX inhibition. The authors combine rational design, multi-step synthesis, spectroscopic characterization, *in silico* ADMET profiling, molecular docking, MM/GBSA binding-free-energy analysis, density functional theory frontier-orbital studies, molecular dynamics simulations, and in vivo anti-inflammatory testing. This multi-layered approach exemplifies how computational tools can help identify promising hybrids, rationalize their interaction with COX 2, and guide the progression of candidates into biological evaluation.

### 2.2. Mechanistic Insight into Vasodilation by Apigenin 7-Glucoside

“The Role of the NO/cGMP Pathway and SKCa and IKCa Channels in the Vasodilatory Effect of Apigenin 7-Glucoside” (Contribution 2) integrates molecular docking with ex vivo pharmacology to elucidate the vascular effects of a plant-derived flavonoid glycoside. Docking simulations indicate favorable binding of apigenin 7 glucoside to endothelial nitric oxide synthase and to small- and intermediate-conductance calcium-activated potassium channels, while mesenteric vascular bed experiments in normotensive and hypertensive rats show endothelium-dependent, dose-related vasorelaxation abolished by high potassium, non-selective K^+^ channel blockade, and combined SKCa/IKCa inhibition. These results link computational predictions to functional data, suggesting that activation of the NO/cGMP pathway and opening of SKCa and IKCa channels underlie the vasodilatory action of apigenin 7 glucoside.

### 2.3. Lipophilicity and Chromatographic Profiling of Thiadiazole Derivatives

In “*In Silico* and RP HPLC Studies of Biologically Active 1,3,4-Thiadiazol-2-yl)-benzene-1,3-diols” (Contribution 3), the authors investigate a series of thiadiazole-containing benzene-1,3-diols using a combination of chromatographic and computational approaches. Lipophilicity parameters are determined experimentally via isocratic HPLC on multiple stationary phases, including octadecyl-bonded, immobilized artificial membranes, cholesterol, and biphenyl columns, while *in silico* descriptors such as logP and logD are calculated to predict pharmacokinetic properties. The study emphasizes that lipophilicity, central to both drug and pesticide behaviour, should be assessed using complementary methods, and that biomimetic chromatographic systems can provide valuable information about membrane affinity and potential distribution.

### 2.4. Benchmarking GNINA and AutoDock Vina for Virtual Screening

The article “High-Throughput, High-Quality: Benchmarking GNINA and AutoDock Vina for Precision Virtual Screening Workflow” (Contribution 4) addresses a key methodological question: how can docking-based virtual screening achieve both throughput and reliability? By comparing AutoDock Vina (v. 1.2.5), a widely used open-source docking tool, with GNINA, an advanced evolution that integrates convolutional neural networks for pose scoring, across ten heterogeneous targets (including metalloenzymes, kinases, and GPCRs), the authors show that GNINA more accurately reproduces experimental binding poses and distinguishes active ligands from decoys, as evidenced by ROC curves and enrichment-factor analyses. The proposed GNINA-based workflow illustrates how deep-learning scoring functions can be incorporated into virtual screening protocols to enhance hit identification and reduce false positives.

### 2.5. Structure-Based Design of Interleukin-6 Inhibitors

“Structure-Based Design of Small-Molecule Inhibitors of Human Interleukin-6” (Contribution 5) explores the possibility of targeting a central pro-inflammatory cytokine with small molecules rather than monoclonal antibodies. The authors perform high-throughput, ensemble docking against conformations obtained from long molecular dynamics simulations of apo human IL 6, informed by prior structural knowledge of contact sites in binary complexes. From the top-scoring ligands, twenty compounds are subjected to functional assays, and one ligand with the second-highest predicted binding affinity achieves approximately 84% inhibition of IL 6-induced STAT3 reporter activity at 10 µM. This study demonstrates the translational potential of structure-based *in silico* screening for cytokine signalling modulation.

### 2.6. Sarolaner Enantiomers and Insect GABA Receptors

Finally, “The Structural Basis of Binding Stability and Selectivity of Sarolaner Enantiomers for Ctenocephalides felis RDL Receptors” (Contribution 6) provides a detailed analysis of how the S enantiomer of the veterinary isoxazoline sarolaner interacts with insect GABA receptors. Homology, fragment-based, and AI-assisted modelling are used to construct wild-type and A285S mutant RDL receptor structures, with SWISS MODEL delivering the most reliable models. Docking, molecular dynamics simulations, and binding-free-energy calculations reveal that the S enantiomer exhibits higher binding affinity and more stable interactions than the R enantiomer, mediated by hydrogen bonding, π–π stacking, and hydrophobic contacts, and that the A285S mutation has limited impact on the binding pocket. These insights support the selective antiparasitic activity of sarolaner and inform future design of isoxazoline-based negative allosteric modulators targeting insect GABA receptors.

## 3. Conclusions

Taken together, the six articles collected in this Special Issue demonstrate several complementary functions of *in silico* methods. They can improve the efficiency of virtual screening, guide the design of new chemical entities, provide structural explanations for biological activity, clarify the molecular basis of selectivity, and support the interpretation of experimental observations. The studies also show that computational research is not restricted to a single discipline: it connects medicinal chemistry, pharmacology, analytical chemistry, molecular biology, structural biology, and agrochemical research.

A further common theme is the importance of methodological complementarity. Docking may be useful for generating binding poses and ranking compounds, but additional approaches may be required to account for receptor flexibility, solvent effects, ligand dynamics, physicochemical properties, pharmacokinetics, toxicity, and environmental fate. Similarly, machine learning predictions are only as reliable as the datasets used for model development and validation. The future of computational discovery will therefore depend not only on increasingly sophisticated algorithms but also on high-quality experimental data, well-defined applicability domains, transparent workflows, and rigorous validation.

The studies included in this Special Issue reinforce the view that *in silico* methods are not substitutes for experimental investigation. Instead, they provide a powerful decision-support framework for formulating hypotheses, reducing experimental workload, prioritizing promising compounds, and interpreting molecular mechanisms. The most reliable discovery strategies will continue to emerge from an iterative cycle in which computational predictions guide experiments and experimental findings refine computational models.

The six contributions in this Special Issue have already attracted attention from the scientific community, as reflected by their citations and views (accessed on 11 August 2026). At the time of this Editorial, the naproxen–azetidinone COX 2 inhibitor study (Article 1) has received 1 citation and 1444 views; the apigenin 7 glucoside vasodilation study (Article 2), 1 citation and 866 views; the thiadiazole lipophilicity study (Article 3), 1124 views; the GNINA/AutoDock Vina benchmarking study (Article 4), 23 citations and 8552 views; the interleukin 6 inhibitor design study (Article 5), 5 citations and 4146 views; and the sarolaner RDL receptor study (Article 6), 4 citations and 1665 views. These metrics illustrate both methodological and applied interest: the docking benchmark and the cytokine-inhibitor design, in particular, have rapidly become reference points for computational workflows and target-specific applications.
